# Supplementary material for: Correcting locomotion dependent observation biases in thermal preference of Drosophila
Source: Sci Rep. 2019 Mar 8;9:3974. doi: 10.1038/s41598-019-40459-z (PMC6408449; doi:10.1038/s41598-019-40459-z)
Supplement: Supplementary file 1 — Supplementary Figures [file 41598_2019_40459_MOESM1_ESM.pdf]

Supplementary Material - Correcting locomotion dependent observation biases in thermal preference of *Drosophila*

Diego Giraldo[1^], Andrea K. Adden[1,2^], Ilyas Kuhlemann[3], Heribert Gras[1] and Bart R. H. Geurten[1*]

[1]Department for Cellular Neurobiology, Institute for Zoology and Anthropology, Georg-August University Göttingen, Germany

[2]current address: Vision Group, Department of Biology, Lund University, Sweden

[3]Department for Biophysical Chemistry, Institute for Physical Chemistry, Georg-August University Göttingen, Germany

* corresponding author: bgeurte@gwdg.de

^ authors contributed equally

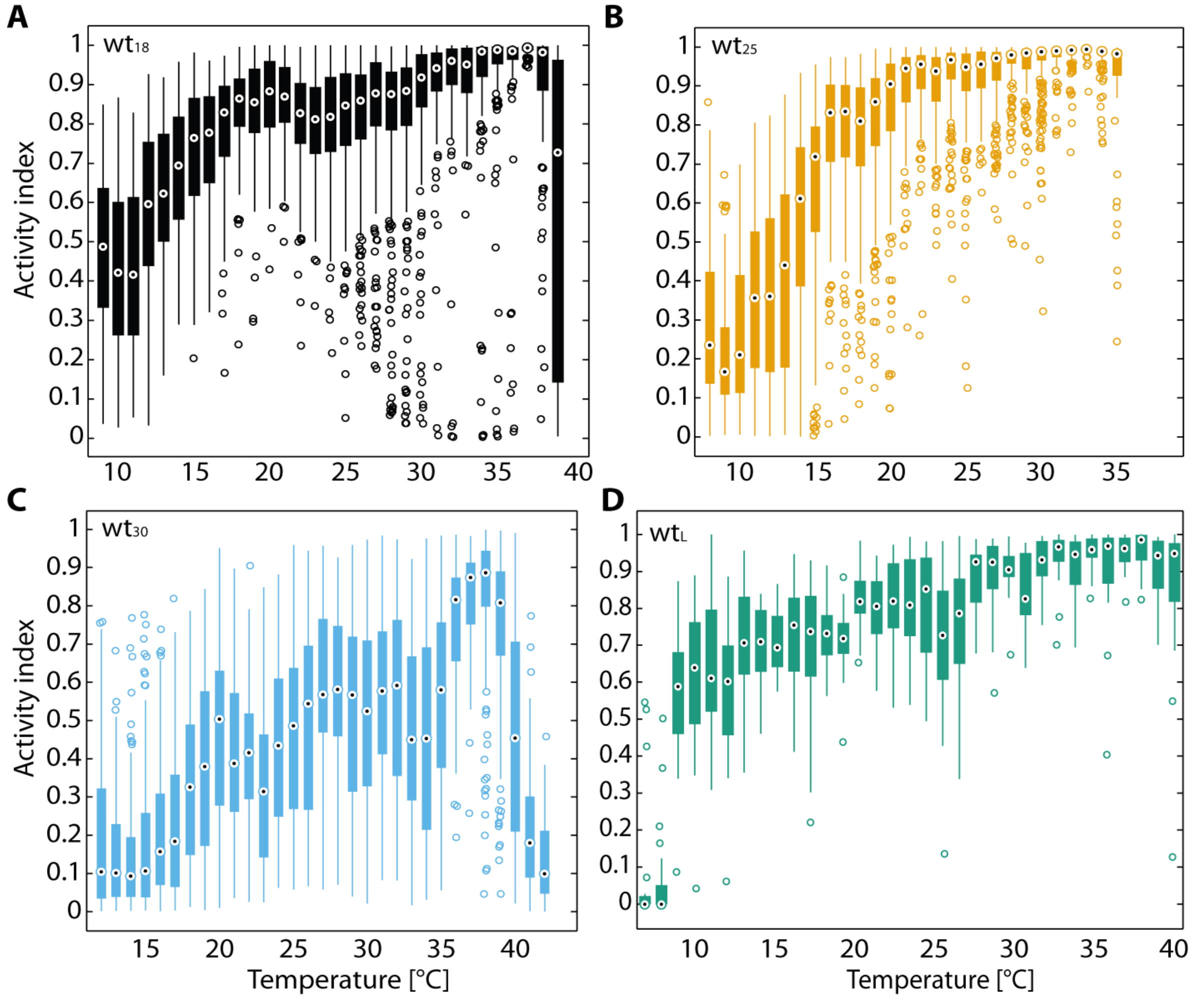

Supplementary Figure 1: Activity of animals during the climbing (adults) / crawling assay (larvae). Movement of more than 10% of the body length per second (0.2 mm/s) was categorised as activity. The index is the amount of time in which the animal was categorised as active / divided by the complete movie duration. The median activity index is noted by the black dot inside a white circle. The two middle quartiles are shown as a coloured box around the median. The whiskers represent either the entirety of the data set or if outliers (empty circles) are present the 1.5 times interquartile range. A) adult CantonS reared at 18°C (wt_{18}) B) adult CantonS reared at 25°C (wt_{25}) C) adult CantonS reared at 30°C (wt_{30}) D) larval CantonS reared at 25°C (wt_L).

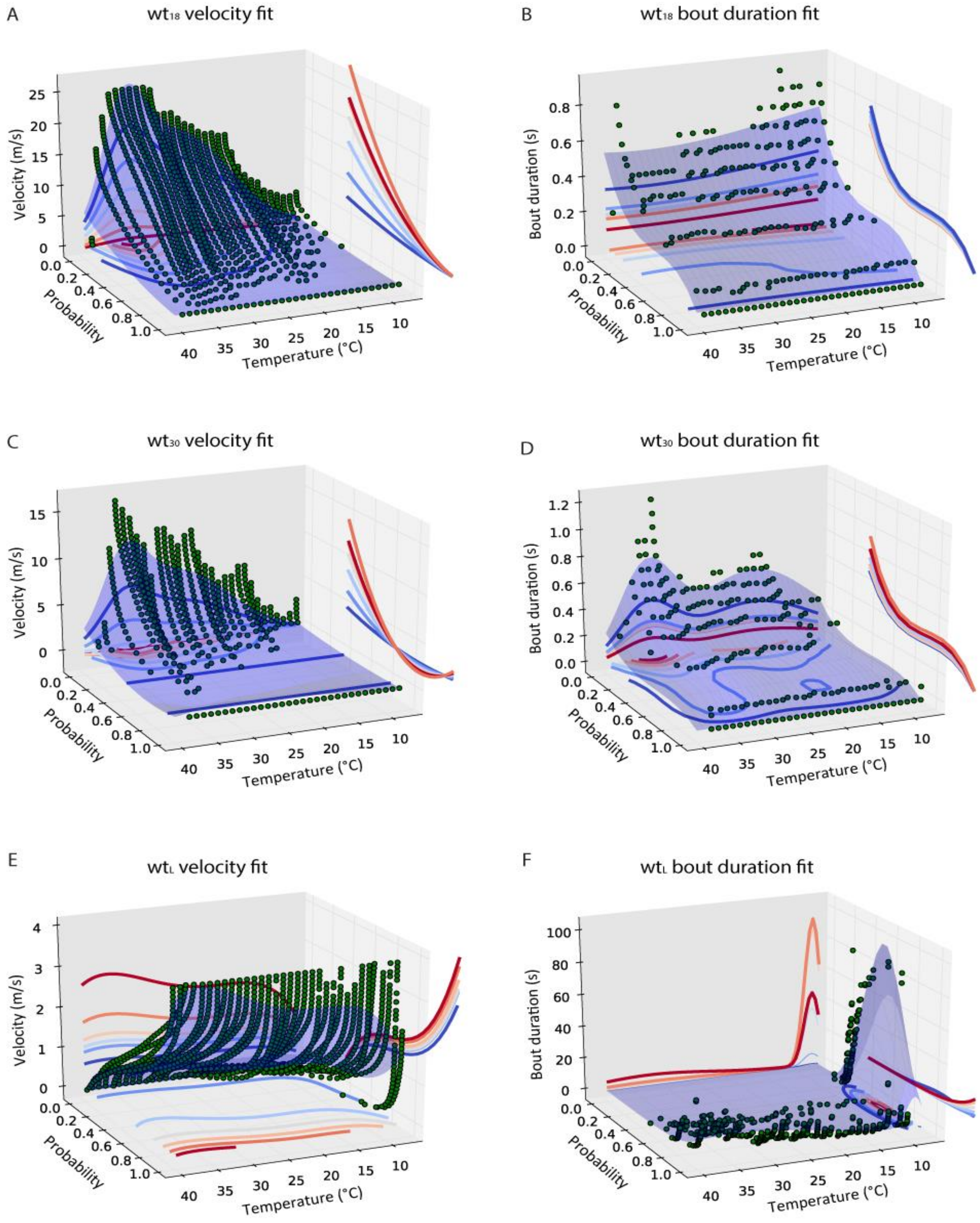

Supplementary Figure 2: A) Probability for a velocity to occur as a function of the ambient temperature for adult flies raised at 18°C (green dots). The shaded area below is the result of the velocity fit function, lines on the wall are the respective projections of the shaded area onto this axis. B) Bout duration fit function shown as in A (18°C raised adult flies). C and D show the same data for adult flies reared at 30°C, while E and F show data of larvae.

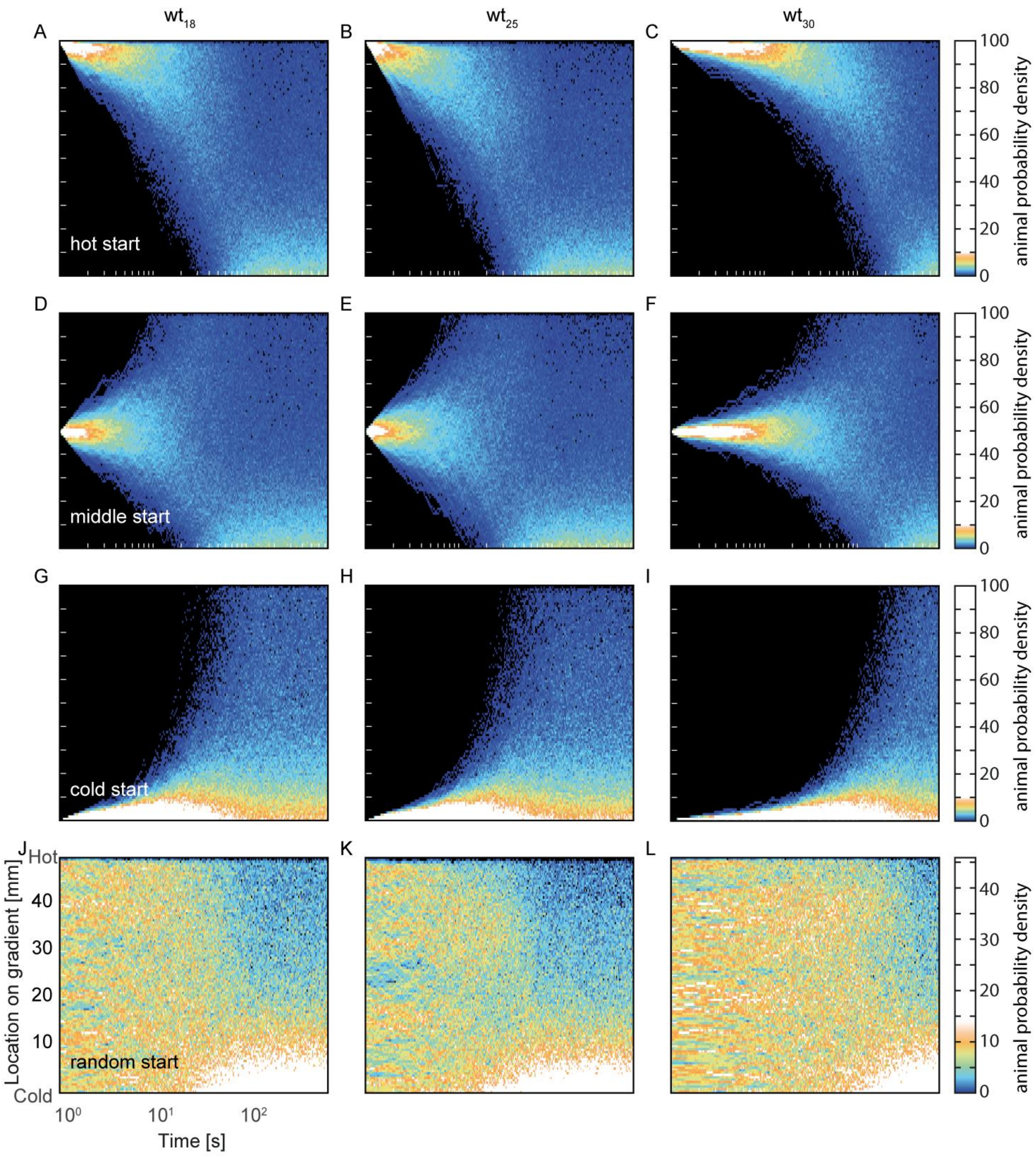

Supplementary Figure 3: Each plot shows the colour coded probability density for a specific combination of T_R and starting position in a temperature gradient over time. The colour bar at the bottom indicates the percentage of flies present at a given point in time and space. Note that the time axis is logarithmic. The three columns each show a different fly group as indicated in the top row. In each row of plots you find a different start condition as indicated in the most left plot. Note that in all cases flies accumulate at the lower right corner, which is evidence of cold aggregation. The colour bar for J-L is different than for A-I.

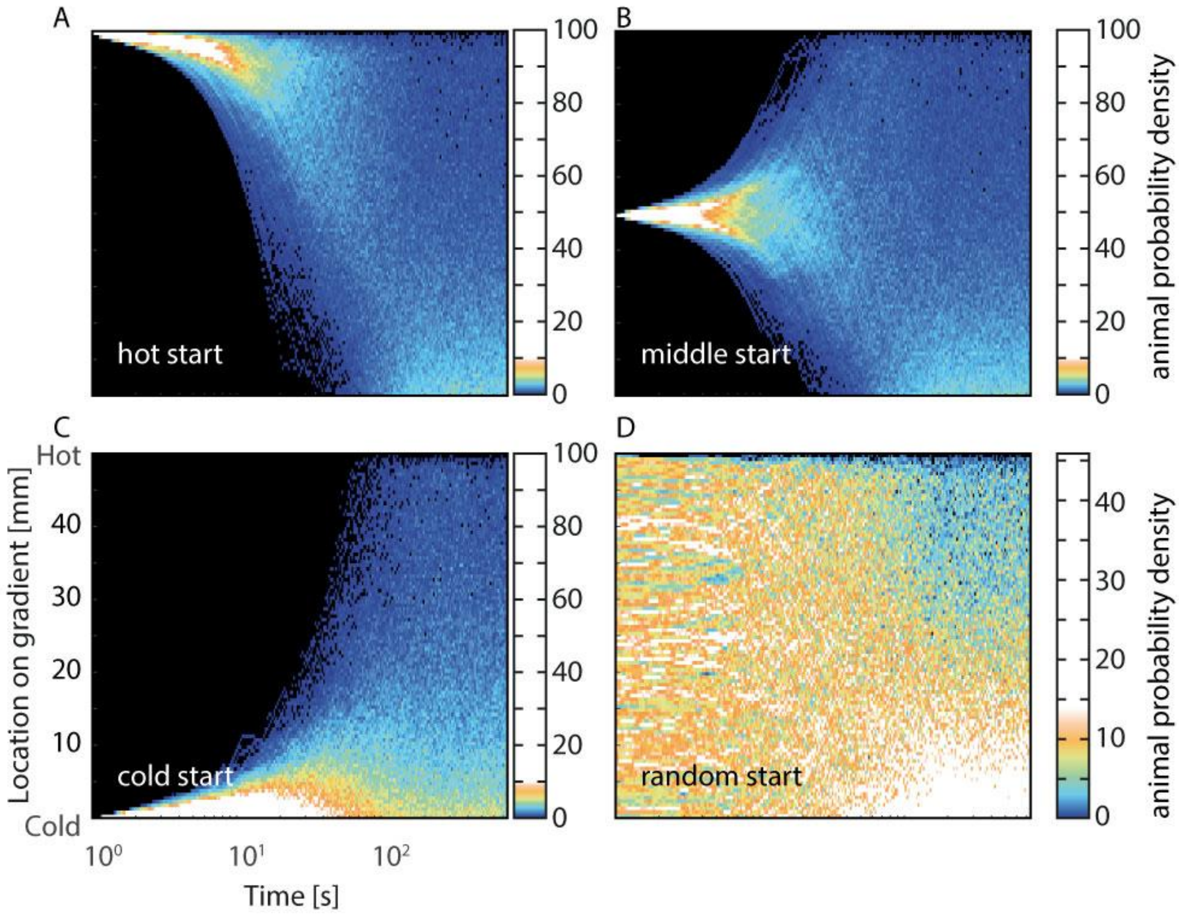

Supplementary Figure 4: Colour coded probability densities for position in a temperature gradient over time for larvae (wt_L). The colour bar at the right indicates the percentage of larvae present at a given point in time and space. Note that the time axis is logarithmic and black indicates 0% even though this may be hard to see in the colour bar given the scale used.. A shows larvae starting at the hot end, while B shows medium temperature start and C cold starts. Random starts are plotted in D. Note that in all cases larvae accumulate at the lower right corner, which is evidence of cold aggregation. The colour bar for D is different than for A-C.

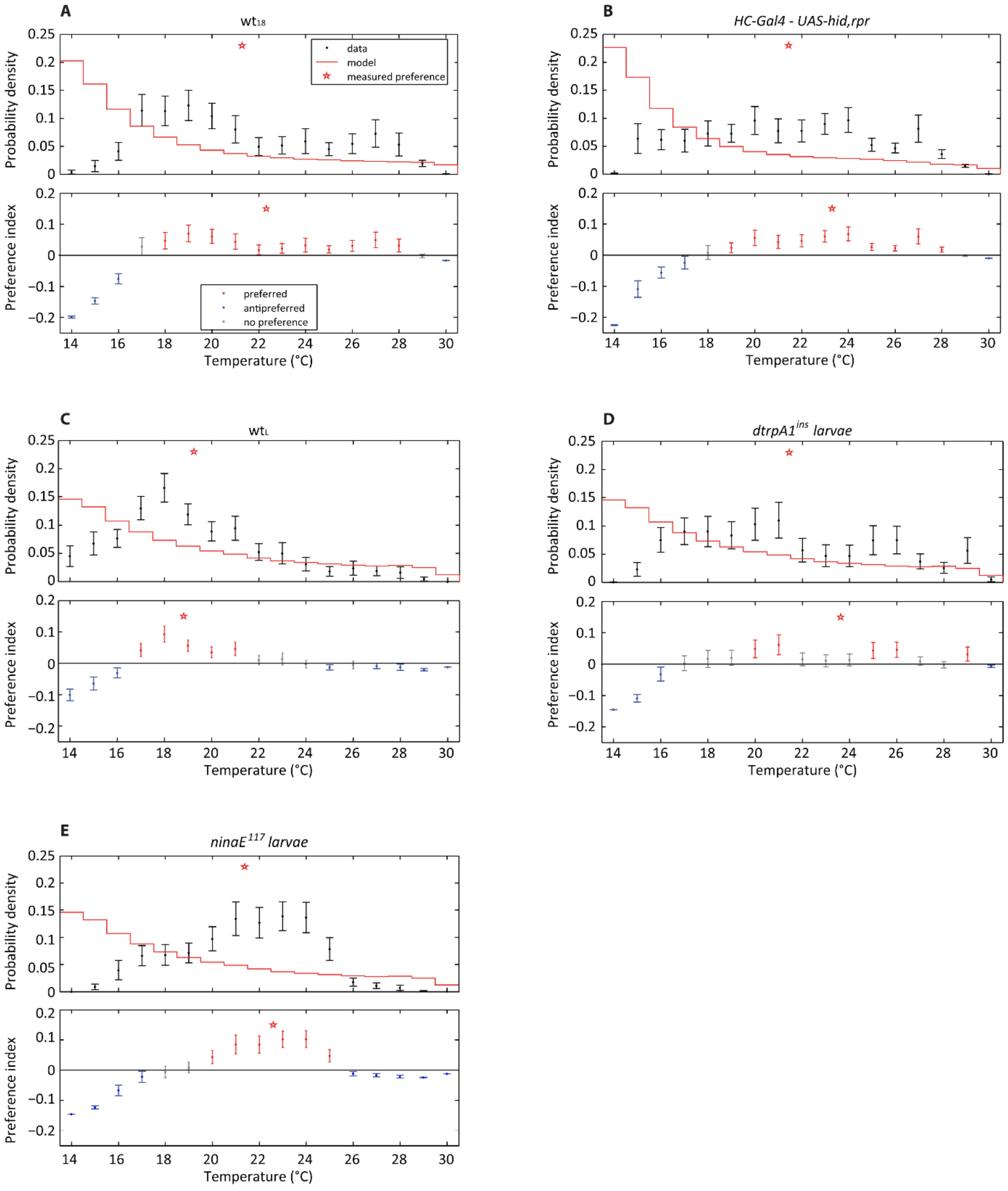

Supplementary Figure 5: Identical to Fig. 3A we plotted here the raw and corrected histograms for all other strains. Strains are indicated above the original histograms. Subplots A and B show data from adult flies, while C-E show larvae results. Each plot is arranged as follows: Upper row shows raw density distribution of animals in a 14°-30°C temperature gradient. Values are median ± 95% confidence interval of the median. The red star indicates the preferred temperature of the tested animals. The red stair plot depicts the result of simulated flies without temperature preference. The lower plot shows the resulting values of the model corrections. Red symbols are indicating temperatures that are significantly more preferred than the null model would predict. Blue values show temperatures that are significantly less preferred. Grey symbols indicates temperatures that are not significantly different from the null model.

As the error bars represent the 95% confidence interval of the median it becomes clear that an error bar that does not span zero shows a significant difference with at least $p<0.05$. (A: wt_{18} n = 50 | B: *HC-Gal4 – UAS-hid,rpr* = 40 | C wt_L =72 | *D: dtrpA1*^{ins} = 60 | E: *ninaE*^{117} =74*)*
